# Parental Reports of Stigma Associated with Child's Disorder of Sex Development

**DOI:** 10.1155/2015/980121

**Published:** 2015-03-31

**Authors:** Aimee M. Rolston, Melissa Gardner, Eric Vilain, David E. Sandberg

**Affiliations:** ^1^Department of Pediatrics and Communicable Diseases, University of Michigan, Ann Arbor, MI 48109-5456, USA; ^2^Department of Human Genetics, David Geffen School of Medicine at UCLA, Los Angeles, CA 90095-7088, USA

## Abstract

Disorders of sex development (DSD) are congenital conditions in which chromosomal, gonadal, or anatomic sex development is atypical. DSD-associated stigma is purported to threaten positive psychosocial adaptation. Parental perceptions of DSD-related stigma were assessed in 154 parents of 107 children (newborn–17 years) questionnaire comprising two scales, child-focused and parent-focused, and three subscales, perceived stigmatization, future worries, and feelings about the child's condition. Medical chart excerpts identified diagnoses and clinical management details. Stigma scale scores were generally low. Parents of children with DSD reported less stigma than parents of children with epilepsy; however, a notable proportion rated individual items in the moderate to high range. Stigma was unrelated to child's age or the number of DSD-related surgeries. Child-focused stigma scores exceeded parent-focused stigma and mothers reported more stigma than fathers, with a moderate level of agreement. Within 46,XY DSD, reported stigma was higher for children reared as girls. In conclusion, in this first quantitative study of ongoing experiences, DSD-related stigma in childhood and adolescence, while limited in the aggregate, is reported at moderate to high levels in specific areas. Because stigma threatens positive psychosocial adaptation, systematic screening for these concerns should be considered and, when reported, targeted for psychoeducational counseling.

## 1. Introduction

Disorders of sex development (DSD) are congenital conditions in which chromosomal, gonadal, or anatomic sex development is atypical. They are classified into three categories based on karyotype: sex chromosome DSD, 46,XY DSD, and 46,XX DSD [1]. DSD differ from other rare conditions, which are often accompanied by significant morbidity and mortality. Although DSD can be associated with life-threatening features (e.g., as in salt-wasting 21-hydroxylase congenital adrenal hyperplasia [2]), most are not. DSD do not generally predict a given level of general physical health or health-related quality of life (HRQoL) across the lifespan and, with limited exceptions, do not spark a medical emergency. Instead, the most serious morbidity associated with DSD is believed to be the lasting emotional and social consequences of being born with atypical sex chromosomes, gonads, or anatomic sex [3–11]. Former patients and advocates commenting on experiences associated with DSD and its clinical management have suggested that “stigma,” and accompanying shame and secrecy, are more strongly predictive of psychological outcomes than the objective severity of the condition or questions about gender [11–13].

Studies of psychological development in other chronic conditions highlight the importance of psychosocial factors, including stigma, in shaping patients' and families' psychosocial adaptation [14–16]. Specifically, with regard to discomfort over the social meaning and acceptance of the medical condition, studies have shown that family members who feel stigmatized by their child's medical condition are at increased risk of experiencing emotional distress and social isolation [17–19]. As medical treatments and disease management have improved, and more pediatric patients with chronic illness are living into adulthood, there is increasing recognition that health care providers need to direct greater attention to psychosocial and HRQoL issues, including stigma, for all pediatric patients with chronic conditions [20].

Despite frequent references to stigmatization as a potent threat to healthy psychological development in DSD [21–23], reports are limited and largely consist of retrospective first person accounts by affected adults [24, 25]. Little is known about the specific forms that stigmatization takes or its frequency/intensity during childhood or adolescence. One recent study that investigated parenting stress and coping patterns of parents of DSD-affected children (*n* = 25; newborn to 10 years) highlights the need for research in this area [26]. Using qualitative research methods, researchers examined: disclosure of child's condition, information received or accessed by parents, sources of support, concerns about forthcoming surgery, concerns about child's future, and suggestions for future service development. Issues such as discussing their child's condition with family/friends, associated stressors, and sources of support (both within and outside their family) were salient topics discussed by these parents. A high proportion of parents (68%) voiced concerns about their child's condition being associated with ridicule or stigma. Study participants were virtually all mothers and the study protocol did not probe for specific sources or the frequency/intensity of the stigma experienced by either the parents or the child [26]. Another recent study reported high levels of posttraumatic stress symptoms (PTSS) in mothers and fathers at the time of their child's DSD diagnosis [27]. Parents were also asked to rate on a 5-point scale the degree they experienced “confusion and disbelief,” “shock,” “shame,” “anger,” “guilt,” “grief,” and “relief” in response to first learning of their child's DSD. Although assessing the cognitive and emotional impact of learning about the DSD, this study did not examine the experience of stigma, in its potentially varied forms at various child ages across childhood and adolescence.

Studies of stigma must take into account that teasing and bullying are relatively common experiences of childhood and adolescence [28, 29]. They can be, but are not necessarily, elicited by distinguishing physical or other characteristics of the targeted person. Among children with medical conditions, perceptions of stigma are influenced by the child's age and peer group [30] and are likely to vary by the informant's relationship to the child [31–33].

In sum, anecdotal accounts and case reports of those affected by DSD and their families raise serious concerns that stigma may serve as a barrier to positive psychosocial adaptation, notwithstanding skilled clinical management. However, as yet, no studies have quantitatively assessed specific, ongoing stigma-related experiences or worries during childhood and adolescence. This is the first study to do so in a relatively large, chart-selected sample in which substantial effort was made to include both mothers and fathers as participants.

This study has two objectives: (1) to quantify parents' perceptions of ongoing DSD-related stigma and assess how these reports vary according to the child's age, characteristics of the child's DSD, and informant relationship (mother or father); and (2) to compare reported stigma by parents of children affected by a DSD to those of parents of children with a different chronic and, similarly not visually obvious, medical condition: epilepsy.

## 2. Methods

### 2.1. Participants and Procedures

Parents and caregivers of DSD-affected children participated in one of two cross-sectional studies. Study 1, a multisite collaboration involving 12 US academic medical centers, focused on developing DSD-specific HRQoL measures for parents of young children. Study 2, involving a single site that was also involved in Study 1, examined the influences of family adaptation and health care delivery on psychosocial adjustment and HRQoL. Households were targeted based on the presence of a child with a DSD (newborn to 6 years, 9 months for Study 1 and 8–17 years for Study 2). DSD diagnosis must have occurred at least 6 months prior to recruitment for Study 1 and one year prior to recruitment for Study 2. To reduce the risk of selection bias, index cases were chart-selected based on ICD-9 diagnosis codes followed by chart review to ensure eligibility. Because of the relatively low incidence, children with sex chromosome DSD (e.g., 45,X/46,XY mixed gonadal dysgenesis) were deliberately oversampled to ensure representation in the study sample. Significant developmental delay (as documented in the medical record) or physical disability accompanying the DSD (e.g., cloacal exstrophy) served as exclusion criteria because of concern that these accompanying features would confound interpretation of findings. The diagnoses of Klinefelter syndrome (47,XXY or its variants) and Turner syndrome (45,X or its variants) were also excluded, as were children born with isolated distal hypospadias because these conditions/presentations are not uniformly classified as DSD [34]. Parent eligibility was restricted to those with functional English literacy, which was assumed unless information in the medical chart suggested otherwise.

Eligible families were mailed a recruitment packet that included an invitation letter signed by a treating provider, informed consent documents, contact information form, “Do Not Contact” postcard, and a postmarked return envelope. Consenting participants were emailed a unique link and log-in password to the online survey. Hard copies of the study questionnaires were mailed to those preferring paper-and-pencil completion or those without Internet access. The majority of participants in both studies (overall, 77.3%) completed the survey online. An honorarium was provided to Study 1 participants only. Both studies were approved by the IRB at each of the recruitment sites.

Overall participation rate was 47.6% (Table 1). Eligible families were classified as “lost to follow-up” if a parent was unreachable (e.g., recruitment packets were returned undeliverable, phone numbers on file were no longer in operation, and/or the family never returned phone calls from the research team). Eligible families were classified as “no response” if they were successfully contacted, but never responded regarding their participation, or if someone from the family agreed to participate, but then never completed any questionnaires.

A total of 154 parents and caregivers (96 women, 58 men) of 107 DSD-affected children (46 girls, 61 boys) participated in one of the two studies (Table 2). Study 1 included 126 parents (64.3% women); Study 2 included 28 parents (53.6% women). Because the vast majority of participants (97.4%) were biological/adoptive parents of affected children, all are hereafter referred to as “mothers” and “fathers.” Participants, across both studies, included a total of 47 mother-father pairs. Ages of the index children (43% reared as girls) ranged from newborn to 16 years (*M* = 4.9 y ± 3.7, median = 4 y; distribution positively skewed and leptokurtic) at the time of the study (Table 3).

### 2.2. Materials

Participants were administered a battery of questionnaires with overlap between Studies 1 and 2. The current analysis is restricted to data obtained from the stigma measure and details from the child's medical chart.

#### 2.2.1. Stigma Measure

As no validated DSD-specific measure of stigma existed prior to this study, a literature review was performed to identify existing instruments that assessed stigma in other chronic pediatric conditions where the condition is similarly not consistently visually obvious. Four questionnaires were identified [30, 35–37]. Each measure included items tapping relevant constructs, but none were thought to be individually adequate to assess the perceptions of stigma in people affected by DSD. Accordingly, items deemed to have face and construct validity for a DSD population were selected from various sources and were combined to create a multiscaled measure assessing parent perceptions of stigma related to DSD. Minor modifications to item wording were made to make items applicable to DSD (e.g., “seizure condition” changed to “urogenital condition”) [30]. All items were scored on 5-point Likert-type scales and were divided, according to content, into two major categories: child-focused (labeled “Part A” on respondent questionnaire) and parent-focused (labeled “Part B”).

The child-focused subscale comprised three items inquiring about the level of agreement (1 = strongly disagree, 5 = strongly agree) with statements regarding thoughts/actions directed towards the affected child. The parent-focused subscale comprised 10 items assessing the consistency of thoughts/actions (1 = never true, 5 = always true) directed towards the parent in relation to their child's DSD. With the exception of substituting the word “urogenital condition,” the child-focused items are identical to those used in the Austin et al. [30] study assessing stigma experienced by children with epilepsy and by their parents. Choosing this scale provided us with the opportunity to compare parent responses in the two patient populations on a set of overlapping items. As such, these items and their corresponding response scale were retained to allow for direct comparison of item responses across patient groups. Comparing parent reports in the DSD population to parents of children with other chronic conditions has rarely been done [27]; most certainly in regards to experiences of stigma, this design is the first of its kind.

We omitted one of the five items in the Austin et al. childhood epilepsy study [30] from our stigma measure due to lack of relevance to the DSD population. A second item in the child-focused subscale, as well as six items from other sources, included in the parent-focused subscale administered to Study 2 participants, was omitted from Study 1 due to overlap with items on other questionnaires. The questionnaire, labeled “Experiences and Reactions,” contained 13 and 20 items in Study 1 and 2, respectively. In addition to the major classification of items as either child or parent-focused, items were further classified to the following a priori scales: (1) perceptions, (2) future worries, or (3) feelings related to their child's condition. The 13 items administered in both studies are listed in Table 4; the seven additional items administered in Study 2 are listed in supplemental Table 1 in the Supplementary Material available online at http://dx.doi.org/10.1155/2015/980121.

In a small pilot arm of Study 2 (not reported here) parents were asked, “Do you think these are important questions to be asking in research like this?” in order to assess the face validity of and relevance to their situation of the Experiences and Reactions questionnaire. The majority of parents (75%) responded “yes” to this open-ended question. Additionally, none reported that any of the items were difficult to understand or answer.

#### 2.2.2. Medical Chart Excerpts

Details from index children's medical charts, including diagnoses, karyotype, gender announcement, current gender of rearing, number of DSD-related procedures (defined as the number of exposures to general anesthesia for DSD-related exploratory or surgical procedures), and degree of genital atypicality (defined as atypicality of the genitals prior to any reconstructive surgery and relative to gender of rearing) were collected (Table 3). Atypicality of genital appearance was assessed based on descriptions of genital appearance at birth in the medical record: the scale for children reared as boys was based on the Quigley scale and ranged from 1 to 7, with 7 representing the most atypical presentation [38]. The scale for children reared as girls was based on the Prader scale and ranged from 1 to 5, with 5 representing the most atypical presentation [39]. Qualified members of the research team reviewed medical chart excerpts and rated each presentation using one of two standardized forms that included both pictorial and verbal anchors for each value on the scale. Medical records were reviewed to determine if there had been uncertainty over the child's gender assignment and/or need for gender reassignment; this variable was coded as follows: (a) gender was announced at birth with no subsequent change, (b) delayed assignment (initial uncertainty over child's sex and with gender assignment being delayed for days, weeks, or months, but without the subsequent need to alter the gender assignment), or (c) gender reassigned (i.e., initial gender announcement/assignment that was subsequently revised). Atypicality of sex chromosomes (relative to gender of rearing, that is, 46,XY girls or 46,XX boys) represented an additional predictor variable; all children diagnosed with sex chromosome DSD (i.e., atypical sex chromosome complement such as 45X,46,XY) were accordingly classified as atypical.

## 3. Data Analysis Plan

### 3.1. Descriptive Statistics: Scale Scores

Total, subscale, and item means (±SD) for the stigma measure were calculated for mothers and fathers separately. Responses ranged from 1 to 5, with “1” representing low levels of agreement/frequency and “5” representing high levels of agreement/frequency of reported stigma. The percentages of parent responses rated “3” or higher for individual items (representing moderate to high concern) were also calculated. The same strategy was followed in reporting the descriptive statistics for the seven items unique to Study 2.

### 3.2. Stigma Scale Reliability

Cronbach's alpha was calculated using the entire sample to assess the internal consistency of the 13-item stigma scale. Alphas were also calculated for the five subscales (child-focused, parent-focused, perceptions, future worries, and feelings).

### 3.3. Comparative Analyses

#### 3.3.1. Stigma Subscales

Paired sample *t*-tests were used to assess differences in reported stigma on child-focused versus parent-focused subscales. A repeated measures ANOVA was used to compare the means of the other subscales (perception, future worries, and feelings).

#### 3.3.2. Cross-Informant Agreement

Correlations between total, subscale, and item scores were performed to assess level of agreement for mother/father pairs. Additionally, paired samples *t*-tests and associated effect sizes (ES) were calculated to assess differences between parents at the scale and item level. Cohen recommends interpreting ES of 0.2 to 0.3 as “small,” around 0.5 as “medium,” and 0.8 or higher as a “large” effect [40]. McNemar's tests were performed to assess the differential frequencies of mothers versus fathers reporting moderate to high concern on individual items (i.e., score of “3” or higher).

#### 3.3.3. Epilepsy Comparison

Independent samples *t*-tests were calculated to examine differences in parent reports between those with children affected by DSD or either new-onset seizures or chronic epilepsy [30]. In the epilepsy study, the chronic epilepsy sample consisted of 173 children (85 girls, 88 boys) aged 9 to 14 years (mean age 11.8 years) and their major caregiving parent, where “with few exceptions, the major caregiver was the mother” (p.475). The new-onset seizures sample consisted of 224 parents of children (116 girls, 108 boys) aged 4 to 14 years (mean age 8.5 years). Again, “with few exceptions, the major caregiver was the mother” (p.475).

### 3.4. Predictors of Stigma

Linear regression analyses were performed using informant demographic characteristics (described in results) to predict stigma. Correlational analyses examined the relationships between child age, genital atypicality at birth, and reported stigma. Partial correlations, controlling for child's age, were used to assess the relationship between the number of DSD-related procedures performed and reported stigma. A one-way ANOVA was used to test for differences in reported stigma across the three DSD diagnostic categories: sex chromosome DSD, 46,XY DSD, and 46,XX DSD.

Finally, independent samples *t*-tests and calculation of ES were used to compare: (1) differences in reported stigma between parents of children with and without sex chromosome atypicality in relation to gender of rearing, and (2) differences in reported stigma between parent reports for children whose gender of rearing was decided at birth and those with delayed decisions. When reported stigma, based on these predictors, differed between mothers and fathers, multiple linear regression analyses were performed with an interaction term (parent gender and the variable of interest) to assess statistical significance. The analyses focusing on DSD diagnostic category also included child gender as a predictor. Total stigma and the five subscale scores were considered as the primary outcomes. These regression analyses were performed using the entire parent sample (mothers and fathers). Violation of the assumption of independence biases against detecting a difference between mothers and fathers; this is, therefore, a conservative manner of assessing the interaction between parent gender and reports of stigma in relation to each of the predictor variables.

Where indicated, set-wise Bonferroni corrections were applied to control for the number of analyses. All statistical analyses were performed using SPSS statistical software (version 22.0), unless otherwise specified.

## 4. Results

### 4.1. Scale Characteristics

#### 4.1.1. Descriptive Statistics: Scale Scores

Total stigma scale scores were generally low (*M* = 1.88 ± 0.62 and 1.67 ± 0.46, for mothers and fathers, resp., on a 5-point scale) (Table 4). A notable proportion, however, reported moderate to high levels of agreement with individual statements (score of “3” or higher), ranging from 4.3% to 61.5% for the mothers and 0% to 45.6% for fathers (Table 5) (Supplemental Tables 1 and 2 for descriptive statistics for the seven unique Study 2 items).

#### 4.1.2. Scale Reliability

The total stigma scale and the five subscales showed moderate to high internal consistency. Cronbach's alpha ranged from 0.65 to 0.86 (Table 4).

#### 4.1.3. Stigma Subscales

Both mothers and fathers reported significantly (*P* < .001) greater child-focused stigma (*M* = 2.28 ± 0.91 and 2.05 ± 0.81) than parent-focused stigma (*M* = 1.76 ± 0.63 and 1.56 ± 0.44) (ES = 0.66 for mothers and 0.75 for fathers). For the other three subscales, both mothers and fathers reported higher scores on items pertaining to future worries and lowest scores for items pertaining to their personal feelings. For mothers, mean differences between all three subscales were statistically significant (*P* < .001). For fathers, ratings for the feelings subscale were significantly lower than ratings for the other two subscales (*P* < .001); ratings for future worries were not significantly different from the perceptions subscale. 

### 4.2. Do Mothers and Fathers Report Different Degrees and/or Forms of Stigma?

#### 4.2.1. Overall Cross-Informant Agreement

Mother-father correlations for the total stigma and subscale scores ranged from *r* = 0.33 to 0.56. Correlations remained statistically significant (*P* < .05) after Bonferroni correction—with exception of the parent-focused and perception subscales (not shown in Table 4). Mothers reported significantly higher total stigma scores than fathers (*P* = .04, ES = 0.33) (Table 4). The same pattern was observed for all subscales, but none achieved statistical significance after Bonferroni correction (Table 4). Differences between parents in reported stigma were of low to moderate effect size, ranging from 0.23 to 0.33. Cross-informant comparisons for seven items unique to Study 2 are available in Supplemental Tables 1 and 2.

#### 4.2.2. Cross-Informant Reports Based on Predictors of Stigma

*Parent Demographic Characteristics*. Linear regression failed to detect significant associations between reported stigma and informant demographic variables (parent age, education, race and ethnicity, or household income); accordingly, these variables were not included in subsequent analyses. 

*Child Age*. Statistically significant correlations between child age and total stigma or stigma subscale scores were not detected. Visual inspection of the scatterplot also did not suggest the presence of any other (e.g., curvilinear) relationship. 

*Number of DSD-Related Procedures*. Statistically significant correlations between number of DSD-related procedures and either the total stigma scale score or the stigma subscale scores (controlling for child's age) were not detected. 

**Figure 1:**
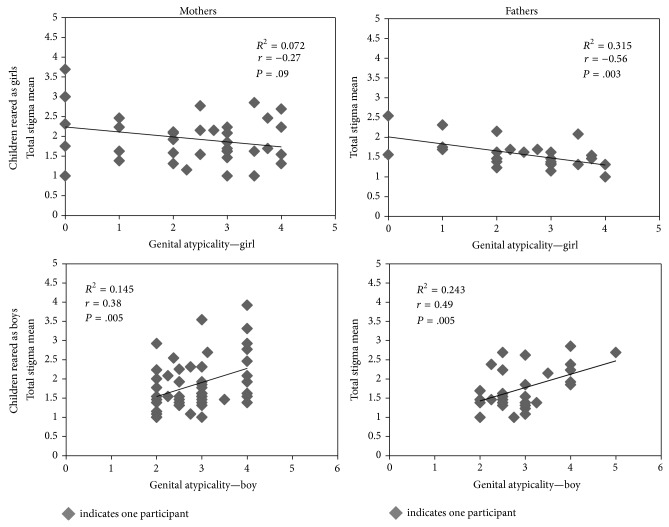
Parent ratings of stigma as a function of child's gender of rearing and degree of genital atypicality at birth. Note: correlation of genital atypicality and stigma subscales not shown, but trends are the same.

*Genital Appearance at Birth*. Degree of atypicality in genital appearance at birth of children reared as girls was negatively correlated with father-reported total stigma; that is, higher atypicality ratings were associated with lower total stigma (*r* = −0.56; *P* = .003). A similar trend was detected for mothers (*r* = −0.27; *P* = .09) (Table 6 and Figure 1). The reverse was true in the case of children reared as boys: atypical genital appearance was positively correlated with total stigma scores reported by mothers (*r* = 0.38; *P* = .005) and fathers (*r* = 0.49; *P* = .005); that is, higher atypicality ratings were associated with higher total stigma ratings (Table 6 and Figure 1). All significant correlations remained so after applying set-wise Bonferroni corrections. The same directionality of correlations was observed for each of the subscales (Table 6). 

*DSD Diagnostic Category*. No significant differences or general trends in mother-reported stigma were found across the three diagnostic subgroups. In contrast, differences in father-reported stigma were observed within subgroup: fathers of children with 46,XY DSD reported greater stigma than did fathers of 46,XX DSD-affected children for the total stigma scale (*P* = .03, ES = 0.85), parent-focused (*P* = .01, ES = 0.99), future worries (*P* = .002, ES = 1.1), and feelings (*P* = .02, ES = 2.0) subscales. To assess whether this apparent difference between mother and father ratings was statistically significant, hierarchical multiple regression analyses, which included a parent gender by diagnostic group interaction term, were performed. Neither parent gender, child diagnosis, nor the interaction term was significant predictors in the full model. To determine whether the observed main effect was confounded with child's gender of rearing, hierarchical multiple regression analyses, including a parent gender by child gender interaction term, were performed. Analyses were restricted to parents of children with 46,XY DSD children due to insufficient numbers of cases reared with a gender discordant with karyotype in the other DSD categories (sex chromosomes DSD and 46,XX DSD) (Table 3). Child gender was a significant predictor of total stigma (*β* = −0.25, *P* = .04, *R* ^2^ change = 0.04), perceptions (*β* = −0.27, *P* = .03, *R* ^2^ change = 0.07), and future worries (*β* = −0.27, *P* = .03, *R* ^2^ change = 0.03). Parent gender and the interaction term were not significant predictors for any of the scales. 

*Atypical Sex Chromosomes in relation to Gender of Rearing*. Mothers of children with atypical sex chromosomes in relation to gender of rearing reported significantly more stigma on the future worries subscale than mothers of children without atypical sex chromosomes (*P* = .01, ES = 0.68). This difference remained statistically significant after a set-wise Bonferroni correction was applied. No statistically significant differences in father-reported stigma as a function of this dimension were found. Multiple linear regression analyses were performed with parent gender, atypical sex chromosomes, and an interaction term with parent gender by atypical sex chromosomes as predictors. Atypical sex chromosomes was a significant predictor of total reported stigma (*β* = 0.19, *P* = .05, *R* ^2^ change = 0.03), perceptions (*β* = 0.19, *P* = .05, *R* ^2^ change = 0.04), and future worries (*β* = 0.25, *P* = .01, *R* ^2^ change = 0.03). Parent gender and the interaction term were not significant predictors for any of the scales. 

*Delays in Assigning Gender of Rearing*. Mothers of children for whom there was a delay in assigning gender reported significantly higher levels of stigma on the child-focused subscale than did mothers of children whose gender of rearing was announced at birth (*P* = .02; ES = 0.62); but, this difference did not remain statistically significant after Bonferroni correction. Similarly, fathers of children with delays in assigning gender of rearing reported significantly higher levels of stigma (after Bonferroni correction) on the feelings subscale compared to fathers of children whose gender was determined at birth (*P* = .004, ES = 0.91). Multiple linear regression analyses were performed with parent gender, timing of gender assignment, and an interaction term for parent gender by timing of gender assignment. Delay in assigning gender of rearing was a significant predictor of child-focused subscale scores (*β* = 0.26, *P* = .03, *R* ^2^ change = 0.01).

### 4.3. Do Parents of Children with DSD Report Different Levels of Stigma Than Parents of Children with Epilepsy?

Austin and colleagues surveyed two epilepsy samples: parents of children with either new-onset seizures or chronic epilepsy [30]. In general, parents of children with DSD reported less stigma than did the parents of children with epilepsy (Table 7). This was true for comparisons with either the chronic or new-onset epilepsy samples (ES range = 0.35 to 0.88). A notable exception to this pattern was observed for the item administered to Study 2 participants only (*n* = 15); parental concern that their child will experience difficulty in finding a romantic/sexual partner. In this particular case, mothers in the DSD sample reported higher levels of stigma than did mothers of children with either chronic (ES = 0.47) or new-onset epilepsy (ES = 0.88); this difference, however, achieved statistical significance (*P* < .001) only in comparison with the new-onset epilepsy sample. All statistically significant differences remained so after applying set-wise Bonferroni corrections.

## 5. Discussion

Much has been written about the experience of stigma surrounding DSD, associated efforts at secrecy, and its potential influence on parental medical decision-making and long-term psychosocial and psychosexual outcomes for DSD-affected people [41–43]. To the best of our knowledge, this study represents the first attempt to quantify stigma associated with DSD as reported by parents during the period of ongoing care. This study is noteworthy in several additional respects: its relatively large sample size and participation based on diagnostic and phenotypic characteristics rather than membership in patient/family support organizations which carries with it the risk of selection bias [44]; targeting of parents of DSD-affected children representing a wide age range, thereby creating the opportunity to examine variation in the experience of stigma as a function of the child's age; inclusion of both mothers and fathers with enough participation from fathers to allow for meaningful comparisons between parents; and inclusion of a comparison group of mothers of children with either new-onset or chronic epilepsy. The experiences of patients with DSD and their families are rarely contrasted with those dealing with different medical conditions [45–48]. Background differences in parents participating in the two studies in addition to their child's diagnosis may have contributed to differences of reported stigma. Nevertheless, a comparison such as this is only very rarely performed in DSD psychosocial research (e.g., [48]) and ideally serves as the impetus for future cross-condition comparisons to address the issue of specificity in the experience of stigma.

Participation rates were lower than desired, but comparable to rates reported for other psychosocial studies in DSD [26, 49]. Comparability with other studies notwithstanding, sampling bias is possible in that those who declined participation could provide different responses than those presented here. This limitation should be balanced against the fact that recruitment was not confined to a narrow range of socioeconomic status, DSD diagnoses, nor child ages. Additionally it has generally been reported that volunteers exhibit better adaptive function than those who refuse [50]. This suggests that rates of stigma reported in this study are possibly conservative estimates of the true prevalence. Lower participation rate in Study 2, relative to Study 1, was not attributable to recruitment protocol differences between the two studies as these were virtually identical, with the exceptions that a monetary incentive was offered only to participants in Study 1. Perhaps more important was that Study 1 recruited parents of younger children (newborn to 6.9 years) and only parents served as informants, whereas in Study 2, target children were older (age 8 to 17 years) and both parents and the affected youths served as participants. The request to directly involve children may have dissuaded some parents from participating and, if so, reinforces the notion that perceived stigma may be a factor in fueling avoidance of situations that might make the DSD more salient. Precisely how such participant selection factors manifest themselves in the reported findings in this and other DSD-related studies remain to be determined.

When considering the experience of stigma, in the aggregate, parents of DSD-affected children reported low levels. A notable proportion, however, reported moderate to high degrees of stigma for select situations, suggesting that specific experiences or concerns can represent salient issues for families and corroborate the retrospective reports of some affected adults [51]. Parents reported greater child-focused than parent-focused stigma, along with higher endorsement of items dealing with future worries related to their child's condition. The future-oriented nature of many of the most strongly endorsed items suggests opportunities for health care providers and patient/family support groups to directly address these and related concerns for the child's future.

The level of agreement between mothers' and fathers' reports of stigma was only moderate, but comparable to parent agreement in the general population for reports of child behavioral and emotional adaptation [52, 53]. Mothers typically reported more stigma than fathers: a pattern matching that observed for mother-father differences in reporting of child's general behavioral and emotional functioning [54]. Both of these observations underscore the importance of independently assessing the mother's and father's concerns over their child's current and future adaptation. Better understanding of the differences between parents in the form or degree of these concerns would potentially inform the shared decision-making process in clinical management.

Each diagnostic DSD category (sex chromosome DSD, 46,XX, and 46,XY) is associated with distinct features and clinical implications for management and HRQoL outcomes, including the varying likelihood of being reared as either a boy or as a girl. In this regard, we found that fathers of children with 46,XY DSD reported significantly more stigma than did fathers of children with 46,XX or sex chromosome DSD. This difference was not observed in mothers' reports; however, the statistical interaction was not significant.

Regression analyses revealed that both mothers and fathers report higher levels of stigma for 46,XY children reared as girls. These analyses were necessarily restricted to parents of children with 46,XY DSD due to either too small sample size (sex chromosome DSD) or lack of variability in gender of rearing (46,XX DSD). Additionally, parents report more stigma for children with delays in determining gender of rearing. These observations require replication because of their relevance to established gender assignment practices. The optimal gender policy, developed by John Money and colleagues in the mid-1950s, replaced reliance on identifying the patient's “true sex” with gender assignment based on the gender predicted to deliver the best combined prognosis for multiple outcomes, most prominently potential for complete sexual functioning, but also psychosocial wellbeing [55]. Following this policy, the recommendation was for 46,XY newborns with rudimentary phallic tissue to be reared as girls with accompanying feminizing genitoplasty [56]. The current finding of higher reported stigma by both mothers and fathers of 46,XY DSD children reared as girls suggests that early psychosocial stresses may discount the anticipated benefits in adulthood of “normalized” genital appearance and function. Of course, this interpretation must be understood in the context of the effect size of the current findings: although statistically significant, gender assignment in 46,XY DSD accounted for only 5% of the variance in reported stigma.

Our findings also suggest that the relationship between degree of atypical genital appearance at birth and reported stigma varies based on the child's gender of rearing. For those reared as girls, the more atypical the initial appearance, the less stigma reported by parents. The opposite was true for parents of children reared as boys: the more atypical the initial genital appearance, the greater stigma reported by parents. This apparent paradox may partly be attributable to differential success of genital surgery in delivering more positive cosmetic results in girls than in boys [57, 58]. It has previously been reported that atypical genitalia in children reared as boys carries a higher psychosocial burden than in girls [59]. Our findings further suggest that these associations are stronger for fathers than for mothers. Research on adults with DSD suggests that men are commonly more concerned about genital appearance, while females are more concerned about function [60, 61]. Again, these relationships are worthy of future investigation to determine, first, whether they can be replicated and, if so, then whether they are modifiable through psychoeducational interventions.

The comparison between parents of children with DSD and parents of children with seizure disorders serves as a reminder that stigma can be a feature of many pediatric chronic conditions, even those for which the stigmatized feature is not obviously visible. Though there may be differences in the degree and specific form that stigma takes across conditions, its presence, as an associated feature, should be considered a threat to positive psychosocial adaptation and HRQoL and be targeted for intervention. Stigma breeds secrecy, shame, and avoidance that can reduce social support, psychological well-being and increase barriers to medical adherence and health-seeking behaviors [62–68].

### 5.1. Clinical Management Implications

Medical condition-related stigma is a well-recognized threat to healthy psychological development and reliable adherence to medical recommendations [69–72]. For these reasons and the findings from this study, serious consideration should be given to routinely assessing whether current or anticipated experiences of stigma are driving parents' decisions (e.g., early genital surgery), influencing parenting styles (e.g., overprotectiveness), and otherwise affecting children's psychological adaptation. The DSD consensus statement emphasizes psychosocial care provided by qualified behavioral health providers as an integral part of clinical management [1]. This implies a model of care in which psychosocial screening and surveillance of predictable challenges to healthy psychological development are proactively addressed—rather than adopting the more traditional consultation model in which medical specialists serve as gatekeepers to behavioral health services [73]. Fully integrated behavioral health services create the possibility of timely and ongoing psychological support (both professional and through peers facing similar circumstances [74]) that challenges unfounded worries and encourages positive adaptive coping strategies if stigmatization is experienced. This integrative approach has precedence in managing other congenital conditions [75]. Pending further development in this area of DSD care, the stigma measure used in this study could be adopted for use in clinic as a tool to assess and guide psychosocial intervention. Our results show that the measure has good internal consistency and has the ability to detect differences between subgroups of patients that one could have intuitively predicted based on previous research.

Might health care providers back away from checking for parents' worries about stigma for fear of iatrogenically introducing these concerns? Findings from this study suggest most parents already have such concerns early in the child's life. Accordingly, neglecting to assess for their presence, and related topics (e.g., sharing information with family and select friends; accessing social support), leaves families to solve problems on their own without the benefit of professional guidance. The example of recommended mental health screening in primary pediatric and adult care amply makes the point that proactive assessment is preferable to crisis management [76, 77]. Only moderate level of agreement between parents in the current study suggests that this assessment should involve both parents. Engaging patients and families in this dialogue should be considered one of the integral steps to achieving patient-centered, comprehensive care.

## Supplementary Material

In the supplemental materials, we provide two tables that give descriptive data regarding the questionnaire items that appeared in Study 2 only. A total of 7 items were analyzed in the same fashion as items analyzed for Tables 4 and 5 in the primary article.

## Figures and Tables

**Table 1:** Participation rates.

	Study 1	Study 2
Total eligible index cases (*n*)	247	67
Lost to follow-up	78 (31.6%)	11 (16.4%)
Declined to participate	44 (17.8%)	17 (25.3%)
No response	35 (14.2%)	22 (32.8%)
Index cases (*n*)	90	17
Participating parents completed	126	28
Participation rate (by index case)^a^	90/169 = 53.3%	17/56 = 30.4%

^a^“Participation rate” = “index cases”/(“total eligible” − “lost to follow-up”).

**Table 2:** Parent demographic characteristics.

	Mothers (*n* = 96)			Fathers (*n* = 58)		
	*n*	%		*n*	%	

Ethnicity						
White	78	81.3		37	63.8	
Other	17	17.7		9	15.5	
Not reported	1	1.0		12	20.7	

	*n*	M (SD)	Range	*n*	M (SD)	Range

Age (yrs)	93	34.9 (7.2)	21–57	52	38.9 (6.2)	26–52
Education^a^	95	5.7 (1.1)	3–7	55	5.9 (1.1)	4–7
Household Income^b^	93	8.4 (3.6)	1–12	55	9.2 (3.4)	1–12

	*n*	%		*n*	%	

Marital status						
Married/living with partner	88	91.7		56	96.6	
Other	7	7.3		1	1.7	
Not reported	1	1.0		1	1.7	

^a^7-point scale: “5” = partial college completion, “6” = college graduation.

^b^12-point scale: “8” = $60,000 to $69,999; “9” = $70,000 to $79,999; “10” = $80,000 to $89,999.

**Table 3:** Index case characteristics (*n* = 107).

	M (SD)	Range	
Age (years)	4.9 (3.7)	0–16	

	*n* (%)		

Gender announcement			
Announced at birth	53 (49.5)		
Delayed assignment	23 (21.5)		
Reassigned	1 (0.0)		
Unknown^a^	30 (28.0)		
Gender of rearing			
Boys	61 (57)		
Girls	46 (43)		

	*n* (%)	Gender assignment	
		boys (*n*)	girls (*n*)

DSD diagnosis			
*Sex chromosome DSD *	*6 (5.6) *	*3 *	*3 *
45,X/46,XY (e.g., mixed gonadal dysgenesis)	6 (5.6)	3	3
*46,XY DSD *	*67 (62.6) *	*56 *	*11 *
Disorders of gonadal (testicular) development	7 (6.5)	6	1
Disorders in androgen synthesis or action	14 (13.1)	5	9
Other (e.g., aphallia, micropenis secondary to panhypopituitarism, and hypospadias)	46 (43.0)	45	1
*46,XX DSD *	*34 (31.8) *	*2 *	*32 *
Disorders of gonadal (ovarian) development	1 (0.9)	1	0
Androgen excess (congenital adrenal hyperplasia)	29 (27.1)	0	29
Other (e.g., Mayer-Rokitansky-Küster-Hauser syndrome, and Müllerian anomaly)	4 (3.7)	1	3
Atypical sex chromosomes relative to gender of rearing	19 (17.8)		

	M (SD)		

Number of DSD-related procedures	2.49 (2.31)		
Genital appearance atypicality at birth relative to gender of rearing			
Boys^b^	2.87 (0.72)		
Girls^c^	2.36 (1.30)		

^a^Insufficient information in medical record to categorize; ^b^7-point scale where 1 = most typical; ^c^5-point scale where 1 = most typical.

**Table 4:** Stigma scale reliability, descriptive statistics, and informant comparisons.

		Total sample				Paired sample			
	Chronbach's *α*	Mothers		Fathers		*n*	*t* ^a^	*P*	ES
		*n*	M (SD)	*n*	M (SD)				
Total stigma score	0.83	96	1.88 (0.62)	58	1.67 (0.46)	47	2.15	.04^*^	0.33
Scales									
Child-focused^b^	0.76	96	2.28 (0.91)	58	2.05 (0.81)	47	1.47	ns	0.23
Parent-focused^c^	0.78	96	1.76 (0.63)	58	1.56 (0.44)	47	1.90	ns	0.33
Subscales									
Perceptions^d^	0.65	96	2.14 (0.82)	58	1.89 (0.72)	47	2.24	.03	0.31
Future worries^e^	0.69	96	2.55 (1.02)	57	2.15 (1.04)	46	1.81	ns	0.30
Feelings^f^	0.86	96	1.63 (0.85)	58	1.39 (0.57)	47	2.02	.05	0.32

(a1) People who know that my child has a urogenital condition treat him/her differently.		96	1.76 (0.88)	58	1.67 (0.85)	47	0.30	ns	0.05
(a2) It really doesn't matter what I say to people about my child's urogenital condition, they usually have their minds made up.		96	2.29 (1.13)	58	2.09 (1.06)	47	0.47	ns	0.08
(a3) In many people's minds, having a urogenital condition attaches a stigma or label to my child.		96	2.80 (1.29)	57	2.42 (1.05)	46	2.40	.02	0.33
(b1) I worry my child will look different from other teenagers or adults because of his/her urogenital condition.		96	2.89 (1.26)	57	2.47 (1.24)	46	1.91	ns	0.28
(b2) I worry my child won't be/isn't able to do things he/she wants to do because of the urogenital condition.		95	2.23 (1.13)	57	1.82 (1.07)	45	1.26	ns	0.23
(b3) I feel that I am odd or abnormal because of my child's urogenital condition.		96	1.58 (1.01)	57	1.30 (0.65)	46	2.19	.03	0.42
(b4) There have been times when I have felt ashamed about having a child with a urogenital condition.		96	1.44 (0.86)	58	1.31 (0.60)	47	0.93	ns	0.15
(b5) I feel self-conscious about my child's urogenital condition.		96	1.94 (1.17)	57	1.53 (0.80)	46	2.18	.04	0.39
(b6^**^) People treat me the way they always have when they find out I have a child with a urogenital condition.		91	2.20 (1.45)	56	2.45 (1.64)	44	−1.02	ns	−0.23
(b7) I feel embarrassed about my child's urogenital condition.		95	1.57 (0.98)	56	1.41 (0.78)	44	0.93	ns	0.15
(b8) People look down on me because I have a child with a urogenital condition.		98	1.28 (0.74)	56	1.16 (0.46)	45	1.96	ns	0.27
(b9) People say negative or unkind things about me behind my back because I have a child with a urogenital condition.		94	1.29 (0.70)	57	1.09 (0.29)	46	2.23	.03	0.37
(b10) I have been excluded from social gatherings because I have a child with a urogenital condition.		94	1.17 (0.62)	56	1.04 (0.19)	45	2.01	.05	0.38

^a^Paired t-test; ^b^consisting of items a1–a3.; ^c^consisting of items b1–b10; ^d^consisting of items a2, a3, and b8;

^e^consisting of items b1 and b2; ^f^consisting of items b3–b5 and b7; ^*^denoting *P* values that remain statistically significant after set-wise Bonferroni correction (or that are <.05 when correction not needed). ^**^Indicating item is reverse scored.

**Table 5:** Frequency of moderate to high concern on individual items reported by mothers and fathers and informant comparisons, ordered by highest percentage in the mother's group.

	Total Sample				Paired comparison^a^	
	Moderate–high concern^b,c,d^				*n*	*P *
	*n*	Mothers	*n*	Fathers		
(b1) I worry my child will look different from other teenagers or adults because of his/her urogenital condition.	96	61.5%	58	40.4%	46	.004^*^

(a3) In many people's minds, having a urogenital condition attaches a stigma or label to my child.	96	57.3%	58	45.6%	46	ns

(a2) It really doesn't matter what I say to people about my child's urogenital condition, they usually have their minds made up.	96	45.8%	58	34.5%	47	ns

(b2) I worry my child won't be/isn't able to do things he/she wants to do because of the urogenital condition.	95	37.9%	57	26.3%	45	ns

(b5) I feel self-conscious about my child's urogenital condition.	96	29.2%	58	15.8%	46	ns

(b6) People treat me the way they always have when they find out I have a child with a urogenital condition.	91	27.5%	57	32.1%	44	ns

(b3) I feel that I am odd or abnormal because of my child's urogenital condition.	96	19.8%	58	10.5%	46	ns

(b7) I feel embarrassed about my child's urogenital condition.	95	15.8%	56	10.7%	44	ns

(a1) People who know that my child has a urogenital condition treat him/her differently.	96	15.6%	58	17.2%	47	ns

(b4) There have been times when I have felt ashamed about having a child with a urogenital condition.	96	12.5%	58	6.9%	47	ns

(b9) People say negative or unkind things about me behind my back because I have a child with a urogenital condition.	94	8.5%	57	0%	46	ns

(b8) People look down on me because I have a child with a urogenital condition.	94	6.4%	56	3.6%	45	ns

(b10) I have been excluded from social gatherings because I have a child with a urogenital condition.	94	4.3%	56	0%	45	ns

^a^McNemar test ^b^Moderate–high concern = response of “3,” “4,” or “5”; ^c^scale for items in Part A: 1 = “strongly disagree,” 2 = “disagree,” 3 = “neither agree nor disagree,” 4 = “agree,” 5 = “strongly agree”; ^d^scale for items in Part B: 1 = “never true,” 2 = “seldom true,” 3 = “sometimes true,” 4 = “usually true,” 5 = “always true.” ^*^Denoting *P* values that remain statistically significant after set-wise Bonferroni correction (or that are <.05 when correction not needed).

**Table 6:** Correlation of reported stigma by mothers and fathers with child's genital atypicality at birth.

	Genital atypicality at birth			
	Children reared as girls		Children reared as boys	
	*r*	*P*	*r*	*P*
*Mothers *				
Total stigma score	−0.27	.09	0.38	.005^*^
Scales				
Child-focused	−0.14	ns	0.41	.003^*^
Parent-focused	−0.28	ns	0.31	.030
Subscales				
Perceptions	−0.26	ns	0.34	.010
Future worries	−0.24	ns	0.45	.001^*^
Feelings	−0.19	ns	0.27	.050
*Fathers *				
Total stigma score	−0.56	.003^*^	0.49	.005^*^
Scales				
Child-focused	−0.53	.006^*^	0.42	.020
Parent-focused	−0.38	ns	0.47	.008^*^
Subscales				
Perceptions	−0.50	.009^*^	0.42	.020
Future worries	−0.44	.030	0.12	ns
Feelings	−0.28	ns	0.31	ns

^*^Denoting *P* values that remain statistically significant after set-wise Bonferroni correction (or that are <.05 when correction not needed).

**Table 7:** Reported stigma in DSD compared to parents of children with epilepsy.

How strongly do you agree or disagree with these comments?	DSD sample, mothers (*n* = 96)	Chronic epilepsy sample^a^ (*n* = 171)			New-onset seizures sample^a^ (*n* = 210)		
	M (SD)	M (SD)	*P* ^c^	ES	M (SD)	*P* ^c^	ES
People who know that my child has a urogenital (seizure) condition treat him/her differently	1.76 (0.88)	2.65 (1.13)	<.001^*^	−0.88	3.00 (1.99)	<.001^*^	−0.81
It really doesn't matter what I say to people about my child's urogenital (seizure) condition, they usually have their minds made up	2.29 (1.13)	2.68 (1.13)	.007^*^	−0.35	3.12 (2.01)	<.001^*^	−0.51
^b^Because of the urogenital (seizure) condition, my child will have problems in finding a boyfriend or girlfriend (husband or wife).	2.47 (1.13)	1.98 (0.93)	.060	0.47	1.50 (1.08)	.001^*^	0.88
In many people's minds, having a urogenital (seizure) condition attaches a stigma or label to my child	2.8 (1.29)	3.28 (1.16)	.002^*^	−0.39	3.70 (2.07)	<.001^*^	−0.52

^a^Reference [30] ; ^b^item appeared in Study 2 only (*n* = 15; child age range 8–16 years) ^c^ *P* value associated with comparison of DSD and epilepsy sample.

^*^Denoting *P* values that remain statistically significant after set-wise Bonferroni correction (or that are <.05 when correction not needed).
