# Supplementary material for: Parental Reports of Stigma Associated with Child's Disorder of Sex Development
Source: Int J Endocrinol. 2015 Mar 31;2015:980121. doi: 10.1155/2015/980121 (PMC4396550; doi:10.1155/2015/980121)
Supplement: Supplementary file 1 — In the supplemental materials, we provide two tables that give descriptive data regarding the questionnaire items that appeared in Study 2 only. A total of 7 items were analyzed in the same fashion as items analyzed for Tables 4 and 5 in the primary article. [file 980121.f1.pdf]

Supplemental Data

Supplemental Table 1. Stigma scale descriptive statistics and informant comparisons for unique Study 2 items

	Total Sample				Paired Sample			
	Mothers		Fathers					
	n	M (SD)	n	M (SD)	n	t[a]	*P*	ES
Because of the urogenital condition, my child will have problems in finding a boyfriend or girlfriend.	15	2.47 (1.13)	13	2.23 (1.01)	11	-0.61	ns	0.22
I worry my child won't get married because of his/her urogenital condition.	15	2.33 (1.05)	13	1.92 (1.12)	11	0.23	ns	0.38
I feel concerned that my child will have/has social problems, like being teased because of his/her urogenital condition.	15	2.33 (1.23)	13	1.92 (0.86)	11	0.21	ns	0.39
I feel protective of my child because of his/her urogenital condition.	15	2.89 (1.26)	13	3.00 (0.91)	11	1.20	ns	-0.10
* I feel comfortable talking to others about my child's urogenital condition.	15	3.53 (1.13)	13	3.31 (1.18)	11	0.71	ns	0.19
I keep my child's urogenital condition private.	15	3.93 (0.96)	13	3.92 (1.19)	11	1.17	ns	0.01
* I feel comfortable talking to others about my child's urogenital condition.	14	2.36 (1.39)	13	2.46 (1.27)	10	-0.51	ns	-0.08

[a] Paired t test * Indicates item is reverse scored

Supplemental Table 2. Frequency of moderate to high concern reported by item and informant comparisons, ordered by highest percentage in the mother's group, for unique Study 2 items.

	Total Sample Moderate – High Concern [a]				Paired Sample	
	n	Mothers	n	Fathers	n	*P*
I feel protective of my child because of his/her urogenital condition.	15	93.3%	13	84.6%	11	ns
I keep my child's urogenital condition private.	15	93.3%	13	92.3%	11	ns
I feel comfortable talking to others about my child's urogenital condition.	15	80.0%	13	76.9%	11	ns
I worry my child won't get married because of his/her urogenital condition.	15	46.7%	13	38.5%	11	ns
Because of the urogenital condition, my child will have problems in finding a boyfriend or girlfriend.	15	40.0%	13	30.8%	11	ns
I feel concerned that my child will have/has social problems, like being teased because of his/her urogenital condition.	15	40.0%	13	15.4%	11	ns
I feel comfortable talking to others about my child's urogenital condition.	14	33.3%	13	38.5%	11	ns

[a] Moderate – high concern = response of "3", "4", or "5"
